# Mapping of Surface-Exposed Epitopes of In Vitro and In Vivo Aggregated Species of Alpha-Synuclein

**DOI:** 10.1007/s10571-016-0454-0

**Published:** 2016-12-27

**Authors:** Leire Almandoz-Gil, Veronica Lindström, Jessica Sigvardson, Philipp J. Kahle, Lars Lannfelt, Martin Ingelsson, Joakim Bergström

**Affiliations:** 1 Department of Public Health and Caring Sciences/Molecular Geriatrics, Uppsala University, Uppsala, Sweden; 2 BioArctic, Stockholm, Sweden; 3 Laboratory of Functional Neurogenetics, Department of Neurodegeneration, Hertie Institute for Clinical Brain Research, University of Tübingen, Tübingen, Germany; 4 German Center of Neurodegenerative Diseases, Tübingen, Germany

**Keywords:** Parkinson’s disease, Dementia with Lewy bodies, Alpha-synuclein, Epitope mapping

## Abstract

Aggregated alpha-synuclein is the main component of Lewy bodies, intraneuronal deposits observed in Parkinson’s disease and dementia with Lewy bodies. The objective of the study was to identify surface-exposed epitopes of alpha-synuclein in vitro and in vivo formed aggregates. Polyclonal immunoglobulin Y antibodies were raised against short linear peptides of the alpha-synuclein molecule. An epitope in the N-terminal region (1–10) and all C-terminal epitopes (90–140) were found to be exposed in an indirect enzyme-linked immunosorbent assay (ELISA) using recombinant monomeric, oligomeric, and fibrillar alpha-synuclein. In a phospholipid ELISA, the N-terminus and mid-region of alpha-synuclein (i.e., 1–90) were associated with phosphatidylserine and thus occluded from antibody binding. The antibodies that reacted most strongly with epitopes in the in vitro aggregates (i.e., 1–10 and epitopes between positions 90–140) also labeled alpha-synuclein inclusions in brains from transgenic (Thy-1)-h[A30P] alpha-synuclein mice and Lewy bodies and Lewy neurites in brains of patients with alpha-synucleinopathies. However, differences in reactivity were observed with the C-terminal antibodies when brain tissue from human and transgenic mice was compared. Taken together, the study shows that although similar epitopes are exposed in both in vitro and in vivo formed alpha-synuclein inclusions, structural heterogeneity can be observed between different molecular species.

## Introduction

The alpha-synucleinopathies are a subset of neurodegenerative diseases that include Parkinson’s disease (PD), dementia with Lewy bodies (DLB), multiple system atrophy (MSA), and the Lewy body variant of Alzheimer’s disease (Goedert 2001). The common pathological feature of the alpha-synucleinopathies is the presence of insoluble cytoplasmic aggregates of alpha-synuclein, termed Lewy bodies and Lewy neurites (Spillantini et al. 1997). In PD, the inclusions are mainly found in dopaminergic neurons in the substantia nigra and other subcortical regions, while in DLB, they are also located in the cerebral cortex (Spillantini et al. 1997, 1998). In MSA, the aggregates are predominantly found in oligodendrocytes and are then referred to as glial cytoplasmic inclusions (Papp et al. 1989).

Alpha-synuclein consists of three distinct structural domains: a lipid-interacting amino-terminus (1–60), a hydrophobic mid-region (61–95), and an acidic carboxyl-terminus with a random coil structure (96–140) (George et al. 1995). It has been believed that alpha-synuclein mainly exists as an intrinsically disordered protein, which adopts an alpha-helical structure upon binding to lipids via its N-terminus and central region (Davidson et al. 1998).

The physiological function of alpha-synuclein remains largely unknown, but increasing evidence suggest that it is involved in neurotransmitter release (Abeliovich et al. 2000; Nemani et al. 2010). For example, alpha-synuclein can promote the formation of the soluble *N*-ethylmaleimide-sensitive factor attachment protein receptor (SNARE) complex by binding phospholipids in the cell membrane with its N-terminus and synaptobrevin-2 with its C-terminus (Burré et al. 2010).

Further implicating alpha-synuclein misfolding in the pathogenesis of neurodegenerative diseases, duplications and triplications of the alpha-synuclein gene (Singleton et al. 2003; Ibáñez et al. 2004), as well as six different point missense mutations have been found to cause either PD or DLB. All the identified point mutations are located in the N-terminal region of the molecule: A53T (Polymeropoulos et al. 1997), A30P (Krüger et al. 1998), E46K (Zarranz et al. 2004), H50Q (Appel-Cresswell et al. 2013), G51D (Lesage et al. 2013), and A53E (Pasanen et al. 2014).

In the alpha-synucleinopathies, alpha-synuclein forms oligomers and protofibrils of increasing sizes, ultimately leading to insoluble fibrils, which constitute the main component of the Lewy body. Mounting evidence suggests that the oligomers are the most neurotoxic molecular species, as they have been shown to cause cytotoxicity both in vitro and in vivo (Danzer et al. 2007; Outeiro et al. 2008; Karpinar et al. 2009; Winner et al. 2011). However, it has not been fully elucidated which parts of the protein are involved in the aggregation process and how well alpha-synuclein aggregates generated in vitro resemble in vivo formed inclusions.

So far, a pair of studies have been performed using antibodies recognizing linear epitopes to characterize structural properties of alpha-synuclein inclusions in the brains of diseased patients (Giasson et al. 2000; Duda et al. 2002). However, no study has systematically compared surface-exposed epitopes of in vitro and in vivo formed alpha-synuclein aggregates. Here, we raised polyclonal immunoglobulin Y (IgY) antibodies against short linear epitopes spanning most of the molecule. These antibodies were used to determine the exposed epitopes of alpha-synuclein monomers and fibrils generated in vitro, as well as inclusions in brain tissue sections from a transgenic (Thy-1)-h[A30P] alpha-synuclein mouse model and from patients with alpha-synucleinopathies.

## Materials and Methods

### Peptide Generation

The immunizing peptides (10–11 amino acids) were custom synthetized at Capra Science Antibodies (Ängelholm, Sweden), and the molecular weight of the peptides was confirmed by mass spectral analysis. The peptides had a purity of about 90% as determined by analytical HPLC. The longer peptides (19–26 amino acids) were custom synthetized at KJ Ross Petersen Aps (Copenhagen, Denmark).

### Antibody Production

Polyclonal IgY antibodies were custom manufactured at Capra Science Antibodies (Ängelholm, Sweden). In short, the synthetic peptides were linked to keyhole limpet hemocyanin and two peptides (100 µg each) per chicken were injected together with Freund’s complete adjuvant (week 0). Two additional boosts (with 100 µg peptide) with Freund’s incomplete adjuvant were performed (weeks 4 and 8). Eggs were then collected and the IgY fraction was purified, followed by an affinity purification step where the antigens were covalently immobilized on an agarose gel and the antibodies were eluted at pH 2.7.

### Production and Purification of Recombinant Alpha-Synuclein

Recombinant wild-type (wt) alpha-synuclein was cloned, expressed, and purified as previously described (Näsström et al. 2011). Mutant alpha-synuclein (A30P, E46K and A53T) was purchased from rPeptide (Athens, GA).

### Generation of Alpha-Synuclein Oligomers and Fibrils

To generate 4-hydroxy-2-nonenal (HNE)-induced alpha-synuclein oligomers, alpha-synuclein (140 µM) in 50 mM sodium phosphate buffer pH 7.4 was mixed with HNE (Cayman Chemical, Ann Arbor, MI) to a final molar ratio of 30:1 (HNE:alpha-synuclein). The samples were incubated quiescently at 37 °C for 24 h, and unbound aldehyde was removed by a Zeba spin desalting column (Thermo Fisher Scientific, Waltham, MA) according to the manufacturer’s instructions. To generate fibrils, wt and mutant alpha-synuclein (140 µM) were incubated in Tris buffered saline (TBS) pH 7.4 in a non-binding polystyrene 96-well plate with agitation for 7 days at 37 °C. The alpha-synuclein fibrils were first pelleted by centrifugation at 16,000×*g*, then re-suspended and washed in TBS and pelleted again by centrifugation. This procedure was performed twice. The fibrillar content of such samples has been verified by atomic force microscopy earlier (Näsström et al. 2011), but to confirm the presence of fibrils in the pelleted material, 1 µl of sample was dried on a glass slide that was stained with Congo red (Sigma-Aldrich, St. Louis, MO). All fibrillar samples exhibited typical apple-green birefringence when viewed under polarized light.

### Indirect Enzyme-Linked Immunosorbent Assay (ELISA)

A 96-well high-binding polystyrene plate (Corning Inc., Corning, NY) was coated with 50 ng antigen (synthetic peptides, monomeric or fibrillar alpha-synuclein) and was incubated overnight at 4 °C. After blocking for 2 h with phosphate buffered saline (PBS) supplemented with 1% bovine serum albumin (BSA, Sigma-Aldrich) and 0.15% kathon (Dow Chemical Company, Midland, MI), the antibodies were incubated at a concentration of 0.5 µg/ml for 1 h in PBS with 0.1% BSA and 0.05% Tween-20 (Sigma-Aldrich). An HRP-bound secondary anti-chicken goat antibody (Jackson ImmunoResearch Laboratories Inc., West Grove, PA) was incubated for 1 h at 0.08 µg/ml in the PBS buffer used for the primary antibody step. The reaction was developed with 100 µl K-Blue Aqueous TMB substrate (Neogen Corporation, Lansing, MI) and stopped with the addition of 100 µl 1 M sulfuric acid (Sigma-Aldrich). Between every step, the wells were washed five times with PBS with 0.1% Tween 20 and 0.0075% kathon. The absorbance was measured at 450 nm using an Infinite M200 Pro microplate reader (Tecan, Männedorf, Switzerland). The signal of the blank (no coated antigen) was subtracted from the sample signals.

### Phospholipid ELISA

1,2-Dioleoyl-sn-glycero-3-phospho-l-serine (DOPS) ELISA Snoopers™ (Avanti Polar Lipids, Alabaster, AL) 8-well strips were blocked with 200 µl/well of blocking buffer [1% fatty acid-free BSA (Sigma-Aldrich) in PBS] at 4 °C overnight. Then, alpha-synuclein samples (50 ng/well) were incubated in PBS for 3 h with gentle shaking at room temperature and washed four times with PBS. The antibodies were incubated at a concentration of 0.5 µg/ml in blocking buffer for 1 h. Following three PBS washes, an HRP-bound secondary anti-chicken antibody (Jackson ImmunoResearch Laboratories Inc.) was incubated for 1 h at 0.08 µg/ml in blocking buffer. After three PBS washes, the reaction was developed with TMB substrate (Neogen Corporation) and 1 M sulfuric acid (Sigma-Aldrich). The absorbance was measured at 450 nm using an Infinite M200 Pro microplate reader (Tecan). The signal of the blank (no antigen) was subtracted from the sample signal.

### Transgenic Alpha-Synuclein Mouse Model

Formalin-fixed and paraffin-embedded brain tissues from homozygous (Thy-1)-h[*A30P*] alpha-synuclein mice (Kahle et al. 2000) were used (*n* = 4; 16, 18, 19, and 22 months of age). The tissue was sagittally sectioned (3 µm). Brain tissue from wt mice (C57BL/6) was used as a negative control (18 months of age). All animal procedures with the (Thy-1)-h[*A30P*] alpha-synuclein mouse have been approved by the regional board of Tübingen, Germany. Animal procedures with the wt mice have been approved by the animal ethics committee of Uppsala, Sweden.

### Human Brain Tissue

The brain samples were obtained from The Netherlands Brain Bank (NBB), Netherlands Institute for Neuroscience, Amsterdam (open access: www.brainbank.nl), project number 848. All the materials were collected from donors from whom a written informed consent for a brain autopsy and the use of the material and clinical information for research purposes had been obtained by the NBB. Substantia nigra samples from four patients [one PD, one DLB, and two Parkinson’s disease with dementia (PDD)] and one neurologically normal control were used (Table 1).

**Table 1:** Summary of information of the patient material used in the study

Case#	1	2	3	4	5
Age	80	80	61	81	80
Sex	F	F	M	M	M
Dx	PDD	DLB	PDD	PD	NN
PMD	5:15	5:00	5:00	4:30	7:00

*Dx* diagnosis, *PD* Parkinson’s disease, *PDD* Parkinson’s disease dementia, *DLB* dementia with Lewy bodies, *NN* neurologically normal, *PMD* postmortem delay

### Immunohistochemistry of Mouse and Human Brain Tissue

Paraffin sections were deparaffinized through ethanol baths of decreasing concentration (99.9–70%) and then washed with distilled water. The sections were pretreated with proteinase K (Life Technologies, Carlsbad, CA) at 50 µg/ml in a buffer containing 10 mM Tris–HCl, 100 mM NaCl, 0.1% Nonidet-P40 (United States Biochemical Corporation, Cleveland, OH) at pH 7.8 and 37 °C for 5 min. Additionally, the human tissue was microwaved in preheated 25 mM citrate buffer for 15 s and allowed to cool down at room temperature for 40 min. All sections were then permeabilized with 0.4% Triton X-100 (Sigma-Aldrich) in TBS for 10 min and treated with 0.3% H_2_O_2_ for 5 min to block endogenous peroxidase reactivity. The sections were blocked with Background Sniper (Biocare Medical, Concord, CA) and incubated at 4 °C overnight with the IgY antibodies at a concentration of 0.5 µg/ml. Detection was performed using a biotinylated secondary anti-chicken (1.5 µg/ml, Jackson ImmunoResearch Laboratories Inc.), followed by incubation with Streptavidin-HRP (1:30, 3310-9, Mabtech AB, Nacka, Sweden). The signal was visualized using the NovaRed substrate kit (Vector Laboratories). The sections were counterstained with hematoxylin (Histolab, Gothenburg, Sweden), dehydrated and mounted with DPX (VWR, Stockholm, Sweden). As a negative control, sections were incubated with secondary antibody alone.

### Neuropathological Analysis of Mouse and Human Brain Tissue

Two independent assessors analyzed immunohistochemically stained sections semi-quantitatively in a blinded manner. The intensity of the staining was scored on a predetermined scale of 0–3 (0 = no signal, 1 = overall faint signal, orange/yellow-colored LB in human tissue, 2 = overall positive signal, red-colored LB in human tissue, 3 = very strong signal, dark red/brown-colored LB in human tissue), and scores were averaged. The differences between mouse and human brain were assessed by a two-way ANOVA and Bonferroni post hoc tests.

## Results

### Characterization of Immunoglobulin Y Antibodies

**Fig. 1:**
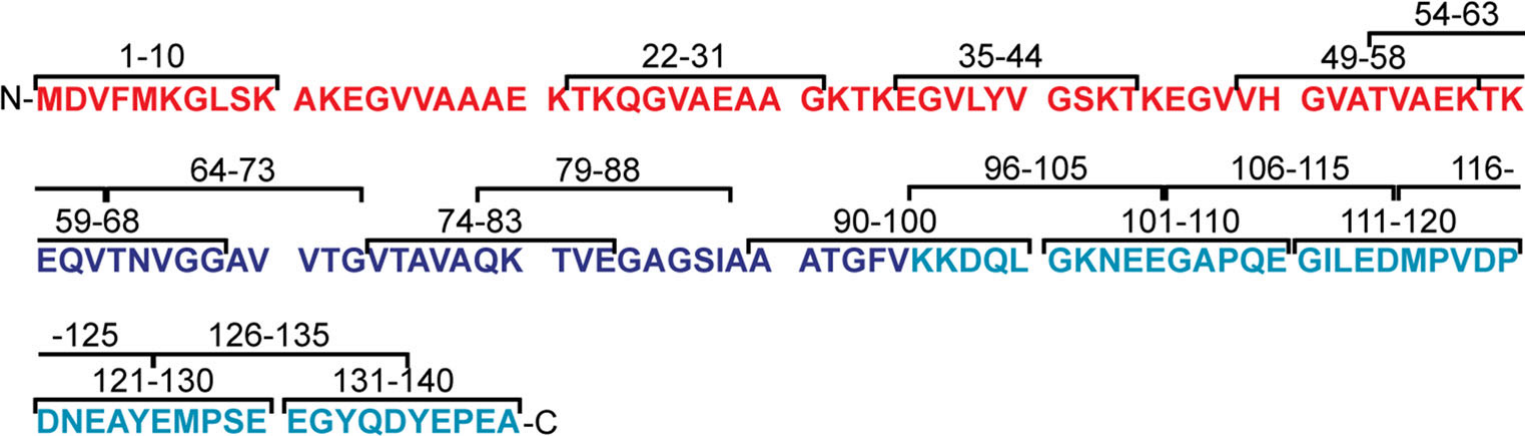
Sequence of immunizing peptides and human alpha-synuclein with its three structural regions: the N-terminus (1–60 *red*), the mid-region (61–95 *purple*), and the C-terminus (96–140 *blue*) (Color figure online)

**Fig. 2:**
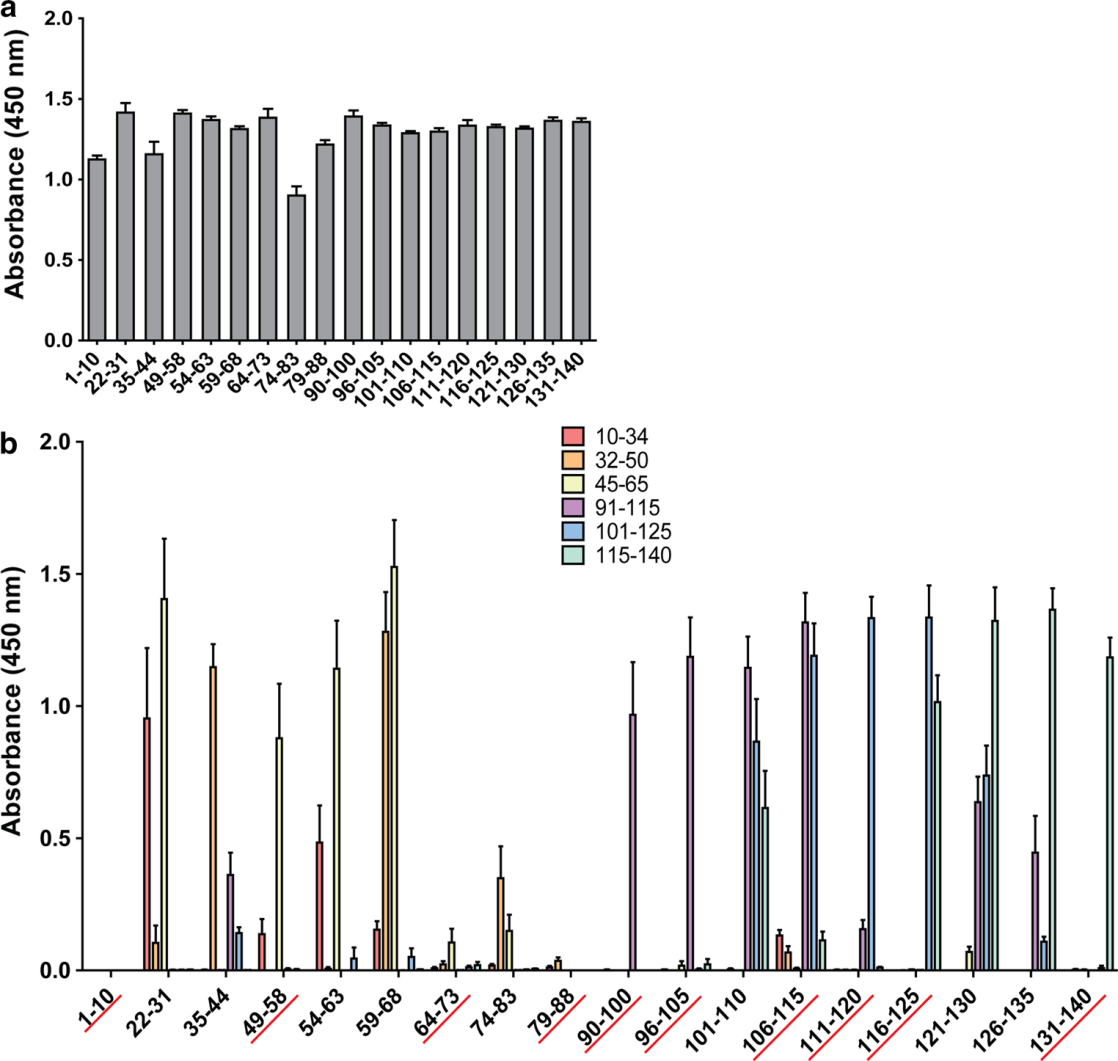
Indirect ELISA characterizing the generated antibodies. The immunizing peptides were coated onto a microtiter plate and analyzed with the corresponding antibody (**a**). Peptides of 19–26 amino acids were coated onto a plate and analyzed with the antibodies to further test their specificity. *Underlined antibodies* showed no cross-reactivity and were selected for the following experiments (**b**). *Error bar*s represent the standard error of the mean (SEM), and each experiment was performed three times with sample duplicates (Color figure online)

In order to determine the surface-exposed epitopes of alpha-synuclein, we generated 18 polyclonal IgY antibodies against short linear peptides spanning most of the alpha-synuclein molecule (Fig. 1). We chose to generate chicken IgY antibodies because of the increased phylogenetic distance between chicken and humans compared to other mammalian species typically used for antibody production (e.g., rabbit) (Hadge and Ambrosius 1984). The sequence identity of chicken alpha-synuclein and human alpha-synuclein is 86.7% compared to 95% between rabbits and humans, which could potentially increase the immunogenicity of the alpha-synuclein peptides. The antibodies were affinity purified against their respective immunizing peptide and showed similar reactivity against them as observed by indirect ELISA (Fig. 2a).

To probe the specificity of the antibodies further, they were used in an indirect ELISA against longer peptides covering different parts of alpha-synuclein (10–34, 32–50, 45–65, 91–115, 101–125, and 115–140) (Fig. 2b). Eight of the antibodies (22–31, 35–44, 54–63, 59–68, 74–83, 101–110, 121–130, and 126–135) also recognized peptides that did not contain their immunizing sequence and therefore were eliminated from the study.

### Indirect ELISA of In Vitro Generated Aggregated Species of Alpha-Synuclein

**Fig. 3:**
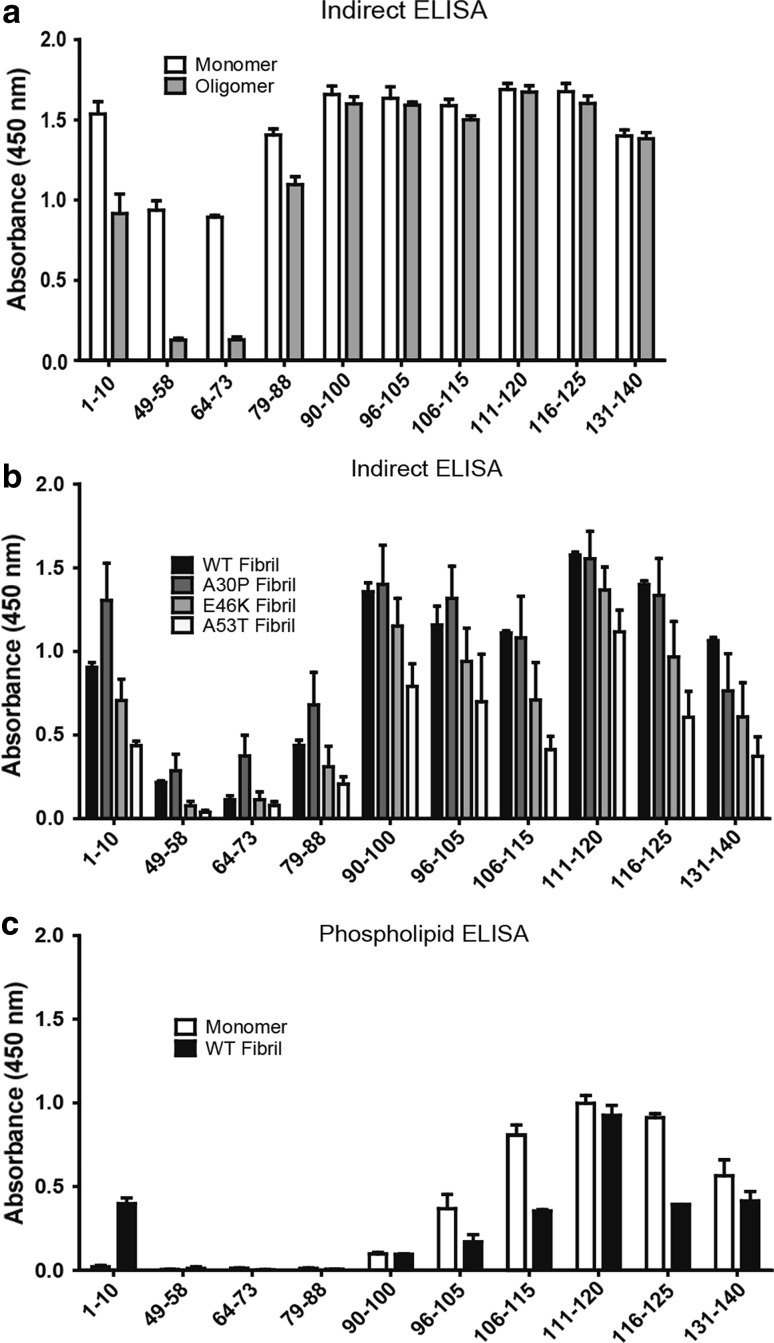
Indirect ELISA. The antibodies were used to analyze surface-exposed epitopes of monomeric and oligomeric alpha-synuclein (**a**) and wt, A30P, E46K, and A53T alpha-synuclein fibrils (**b**). Monomeric and fibrillar alpha-synuclein were incubated in a precoated phosphatidylserine microtiter plate for an indirect phospholipid ELISA (**c**). The *error bars* represent the SEM. Each experiment was performed three times (except in the phospholipid ELISA; *n* = 2) with sample duplicates

First, we mapped the surface-exposed epitopes of recombinant monomeric and oligomeric alpha-synuclein (Fig. 3a). All antibodies recognized monomeric alpha-synuclein, but 49–58 and 64–73 reacted to a lesser extent. The N-terminal antibody 1–10 reacted with oligomers, while 49–58 did not. In the mid-region, 64–73 was negative against oligomers and 79–88 was positive. The C-terminal antibodies (90–140) all recognized oligomeric alpha-synuclein.

Point mutations in the alpha-synuclein (*SNCA*) gene enhance aggregation and cause early-onset PD or DLB (Polymeropoulos et al. 1997; Krüger et al. 1998; Conway et al. 2000; Zarranz et al. 2004; Greenbaum et al. 2005). We generated fibrils consisting of either wild-type (wt) protein or the three mutants (A30P, E46K and A53T) and analyzed them using indirect ELISA (Fig. 3b). The overall reaction pattern for the different fibrils was similar, where the antibody against 1–10, as well as the C-terminal (i.e., 90–140) antibodies showed the highest signals. Overall, the strongest immunoreactivity was observed for wt and A30P fibrils, whereas the lowest signals were seen with the A53T fibrils.

Alpha-synuclein interacts with anionic phospholipids in the neuronal membrane, and the binding has been suggested to be facilitated by the N-terminal part of the protein (Davidson et al. 1998). To determine the lipid-binding ability of the various alpha-synuclein species and to discern the membrane-associated epitopes, an indirect phospholipid ELISA was used. Monomeric and fibrillar (wt) alpha-synuclein were incubated on a plate pre-coated with phosphatidylserine, and the interaction was probed with the antibodies (Fig. 3c). Antibodies against the C-terminus (96–140) reacted with monomeric alpha-synuclein, and to a lesser extent fibrils, with the exception of the antibody 111–120 that reacted to fibrils equally well. In contrast, the antibodies against the N-terminus and mid-region (i.e., 1–95) did not react with monomers or fibrils (with the exception of 1–10 that recognized fibrils), indicating that this region of the molecule was indeed interacting with phosphatidylserine.

### Immunohistochemistry of Mouse and Human Brain

Next, we wanted to analyze exposed epitopes of in vivo deposited alpha-synuclein aggregates by performing immunohistochemistry with the antibodies on formalin-fixed and paraffin-embedded tissue sections of (Thy-1)-h[A30P] alpha-synuclein transgenic mouse brain and sections from the substantia nigra of PD, PDD, and DLB patients. We performed a proteinase K treatment to digest non-fibrillar alpha-synuclein (Giasson et al. 2001; Miake et al. 2002; Neumann et al. 2002) and to ensure that the aggregates observed in both mouse and human tissue were comparable.

**Fig. 4:**
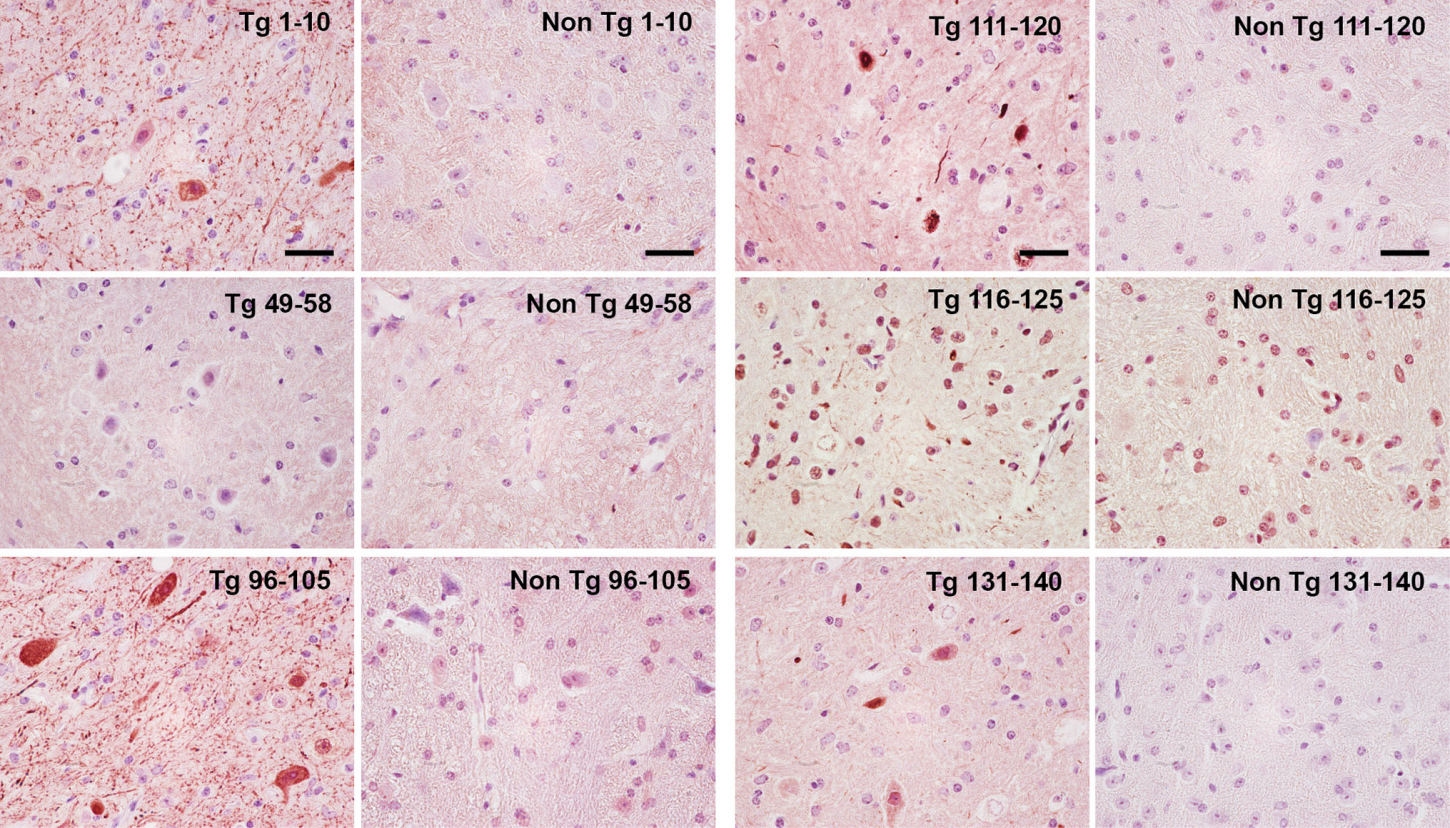
Representative images of immunohistochemical staining of (Thy-1)-h[A30P] alpha-synuclein transgenic (Tg) mouse cerebellum. Non-Tg (non-transgenic). *Scale bar* 20 µm

The (Thy-1)-h[A30P] alpha-synuclein transgenic mouse model presents cognitive decline at 12 months of age, whereas PD-like motor symptoms can be observed at 17 months (Kahle et al. 2000; Schell et al. 2009). Brain sections from four mice at or after onset of motor symptom were analyzed. Alpha-synuclein aggregates could be observed in the cerebellum, mid-brain, pons, medulla and to a lesser extent in the cortex. Four types of alpha-synuclein aggregates could be distinguished: (1) small, dotted grain-like intracellular aggregates, which were the most abundant in all brain regions; (2) Lewy-neurite-like elongated structures, which sometimes accompanied the grain-like staining; (3) Lewy body-like inclusions, which could occasionally be observed, most frequently in the cerebellum, and (4) diffuse nuclear and cytoplasmic accumulation of alpha-synuclein, which was predominantly observed in the cerebellum and mid-brain (Fig. 4). The highest immunoreactivity was observed with the C-terminal epitope 96–105. The N-terminal antibody 1–10 and C-terminal antibodies 90–100, 106–115, 111–120, 116–125, and 131–140 also stained the described structures, with variable intensities. The antibody against 131–140, in particular, showed less immunoreactivity. No staining was observed with the antibodies 49–58 and 64–73, and very faint staining with 79–88 could be observed.

**Fig. 5:**
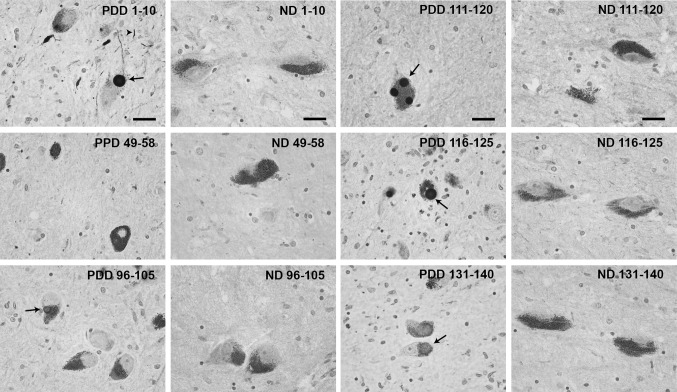
Representatitve images of immunohistochemichal staining of substantia nigra brain tissue from a PDD patient and non-diseased (ND) brain. *Arrows* indicate Lewy bodies and *arrowheads* indicate Lewy neurites. *Scale bar* 20 µm

In the human PD, DLB, and PDD brain tissue, 1–10 and the C-terminal antibodies 111–120 and 116–125 showed the highest reactivity and stained both Lewy bodies and Lewy neurites (Fig. 5). The rest of the C-terminal antibodies (90–100, 96–105, 106–115, and 131–140) produced a weaker staining of inclusions. No immunoreactivity of Lewy body pathology was detected with antibodies recognizing epitopes 49–58, 64–73, and 79–88, which correspond to the latter part of the N-terminus and mid-region of alpha-synuclein. No differences were observed amongst tissue from patients with different alpha-synucleinopathies (i.e., PD, DLB, and PDD).

**Fig. 6:**
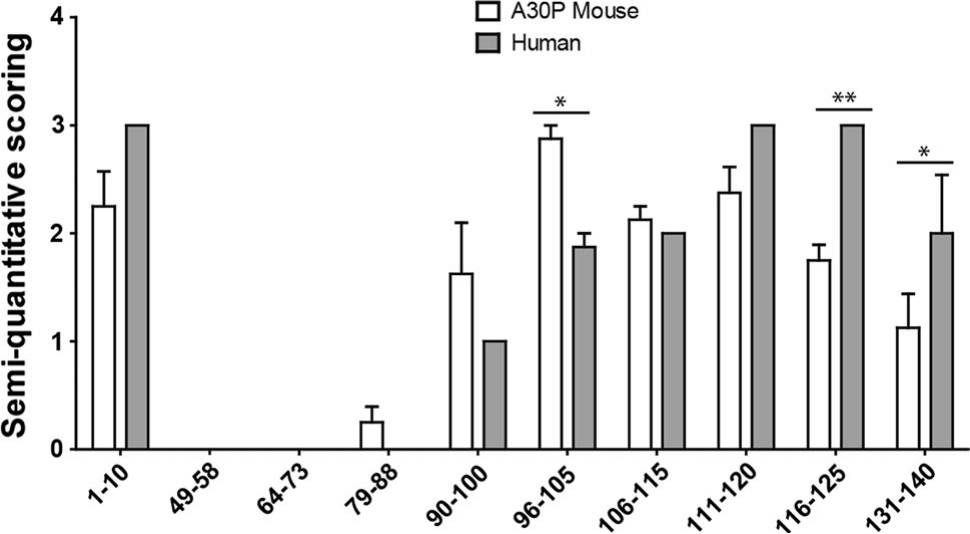
Semi-quantitative assessment (0–3) of the intensity of the immunostaining of four (Thy-1)-h[A30P] alpha-synuclein transgenic mouse brains and four alpha-synucleinopathy patients. *P < 0.05; **P < 0.001, two-way ANOVA followed by Bonferroni post hoc test, *error bars* represent the standard error of the mean (SEM)

The staining was semi-quantitatively analyzed by visual scoring between 0 and 3 by two blinded independent assessors (Fig. 6). Significant differences were observed between the intensity of the staining of the transgenic mice and the diseased human brains by the 96–105, 116–125, and 131–140 antibodies.

## Discussion

In Lewy body disorders, the alpha-synuclein protein loses its native structure and aggregates as insoluble Lewy bodies and Lewy neurites (Spillantini et al. 1997). The fibrillation of alpha-synuclein has been studied to a great extent in vitro. However, a wide range of heterogeneous structures is formed during the aggregation of recombinant alpha-synuclein, and it is uncertain how well they reflect the aggregates formed in vivo. With the aim to identify similarities and differences of in vitro and in vivo formed alpha-synuclein aggregates, we generated 18 polyclonal antibodies against peptides covering most of the alpha-synuclein sequence. However, when the specificity of the antibodies was tested, we observed that eight of the antibodies also reacted with alpha-synuclein epitopes outside their immunizing sequence and hence they were omitted from the study due to the unspecific binding. In previous studies (Giasson et al. 2000; Duda et al. 2002), antibodies recognizing linear epitopes have been used to characterize alpha-synuclein aggregates, but compared to the present investigation, fewer epitopes were studied and mostly human material was analyzed.

In agreement with the structure of a disordered protein, recombinant monomeric alpha-synuclein reacted with all the antibodies; however, the 1–10 and all C-terminal antibodies showed higher reactivity. In vitro generated fibrils of alpha-synuclein have been shown to contain a beta-sheet-rich core (Heise et al. 2005; Chen et al. 2007), spanning approximately residues 35–96 as observed by nuclear magnetic resonance spectroscopy, although the exact length of the core varies amongst studies (Vilar et al. 2008; Tuttle et al. 2016). In agreement with this model, the fibrils analyzed by the ELISA showed that epitopes 49–58 and 64–73 were not accessible, and presumably hidden in the core of the fibril structure. The exposed epitopes in the oligomers were similar to those in the fibril, with the exception of 79–88, which was positive in the oligomers but only weakly reactive in the fibrils. The alpha-synuclein mutations A30P, E46K, and A53T have been shown to oligomerize faster than the wt protein in vitro (Conway et al. 2000; Greenbaum et al. 2005). Furthermore, atomic force microscopy studies indicate that the fibrils generated with these alpha-synuclein mutations may exhibit structural heterogeneity compared to fibrils formed by wt protein (van Raaij et al. 2006). However, in the current study, the surface-exposed epitopes of A30P, E46K, and A53T fibrils did not differ to any large degree; suggesting that although mutant alpha-synuclein may aggregate faster than wt protein, the fibrils formed exhibit a similar structure.

Alpha-synuclein exists in equilibrium between a cytosolic and a membrane bound state in vivo (Davidson et al. 1998). The seven imperfect repeats (KTKEGV) located in the N-terminus exhibit a variation in hydrophobicity that is typical of the amphipathic lipid-binding α-helical domains of apolipoproteins (George et al. 1995). As lipid binding has been suggested to be an important factor for the physiological function of alpha-synuclein, we used a phospholipid ELISA assay to probe which epitopes interact with phospholipids in different molecular species of alpha-synuclein. We chose to use a lipid ELISA assay based on phosphatidylserine due to the fact that this is a common anionic lipid of eukaryotic cellular membranes and is known to bind to alpha-synuclein (Davidson et al. 1998; Pranke et al. 2011; Zarbiv et al. 2014). For monomeric alpha-synuclein, only C-terminal epitopes (i.e., 96–140) were accessible, and this indicates that the N-terminus and the mid-region of the protein were indeed lipid bound and hence not available for antibody binding. Interestingly, wt fibrils also showed a strong binding to phosphatidylserine, and similar to monomeric protein, the C-terminus was most accessible to antibody binding. This suggests that the N-terminus and the mid-region (i.e., 1–95) are responsible for the interaction with phosphatidylserine, which is consistent with the previously mentioned lipid-binding domain of alpha-synuclein. The surprising finding that the N-terminal part of the fibrils (i.e., 1–10) was somewhat accessible could most likely be explained by the fact that as several protofilaments constitute a single fibril, not all their N-terminal parts need to be associated with lipids in order to facilitate binding. It should be noted that in the phospholipid ELISA, the phosphatidylserine molecules are forming a planar layer, and since alpha-synuclein is highly sensitive to membrane curvature (Middleton and Rhoades 2010), the exposed epitopes could potentially vary when bound to cellular membranes and lipid vesicles.

To the best of our knowledge, no study has compared alpha-synuclein inclusions in human and mice brain tissue. Therefore, we used our novel antibodies to analyze the alpha-synuclein immunoreactivity in the brain of a tg mouse model overexpressing human A30P mutant alpha-synuclein (Kahle et al. 2000), as well as in substantia nigra specimens from patients with alpha-synucleinopathies. The proteinase K treatment is routinely used to assess fibrillar alpha-synuclein in tissue (Kramer and Schulz-Schaeffer 2007; Tanji et al. 2010), and it allows us to observe structures that are similar in compactness and morphology. In the alpha-synuclein transgenic mice, the most abundant pathology was found in the brainstem (Neumann et al. 2002; Freichel et al. 2007), where small aggregates were observed, only occasionally accompanied by larger, more compact inclusions, similar to those found in human tissue. Nuclear and cytoplasmic staining of neurons was also frequently detected with the most reactive antibodies. In both human and mouse tissue, the strongest reactivity was observed with antibodies against the 1–10 epitope in the N-terminus and the C-terminus. However, we found differences in the intensity of the staining between these tissue types for antibodies 96–105, 116–125, and 131–140. Taken together, these findings indicate that there might be structural differences between the aggregates formed by human alpha-synuclein in mice and human brains. Generally, a greater variability of the overall amount of alpha-synuclein pathology was observed amongst the mice, but it did not affect the general staining pattern of the antibodies.

A previous study using monoclonal antibodies that recognized epitopes between 87 and 140 and two polyclonal antibodies that recognized 2–12 (SNL-4) and 104–119 (SNL-1) showed strong staining of Lewy bodies and neurites in the substantia nigra of PD and DLB patients (Giasson et al. 2000). The results with the SNL-4 antibody are comparable to the observations of the 1–10 antibody in the present study and the SNL-1 is similar to the antibody 111–120, which recognized abundant Lewy body pathology. Additionally, Kovacs et al. (2012) generated and characterized a monoclonal antibody (5G4), which specifically recognizes amino acids 44–57 and strongly labels alpha-synuclein pathology in human brain tissue. In contrast, our antibody against epitope 49–58, which recognizes a similar epitope, failed to detect any alpha-synuclein pathology in both the mice and the human tissue. This suggests that the lack of reaction with one of the IgY antibodies does not necessarily mean that the entire sequence used to produce that antibody is hidden. The difference in immunoreactivity could most likely be explained by the fact that 5G4 recognizes a slightly different epitope.

Taken together, the present study shows that some parts of the protein always appear to be exposed on the surface; specifically, the beginning of the N-terminus and the entirety of the C-terminus were recognized by the IgY antibodies in both in vitro monomeric and aggregated samples, as well as in brain sections. On the other hand, with the exception of the native protein, most of the mid-region of aggregated alpha-synuclein was consistently occluded in aggregated forms, which is in agreement with the previously mentioned fibril core. However, the finding that C-terminal epitopes were exposed differently in tg mice and human tissue also suggests that subtle structural differences can exist in the alpha-synuclein aggregation species formed in different organisms.
